# Early Warning Signals in Phase Space: Geometric Resilience Loss Indicators From Multiplex Cumulative Recurrence Networks

**DOI:** 10.3389/fphys.2022.859127

**Published:** 2022-05-04

**Authors:** Fred Hasselman

**Affiliations:** Behavioural Science Institute, Radboud University, Nijmegen, Netherlands

**Keywords:** multiplex recurrence networks, multivariate time series analysis, resilience loss indicator, multidimensional recurrence quantification analysis, complex adapt system (CAS), process monitoring, transition network

## Abstract

The detection of Early Warning Signals (EWS) of imminent phase transitions, such as sudden changes in symptom severity could be an important innovation in the treatment or prevention of disease or psychopathology. Recurrence-based analyses are known for their ability to detect differences in behavioral modes and order transitions in extremely noisy data. As a proof of principle, the present paper provides an example of a recurrence network based analysis strategy which can be implemented in a clinical setting in which data from an individual is continuously monitored for the purpose of making decisions about diagnosis and intervention. Specifically, it is demonstrated that measures based on the geometry of the phase space can serve as Early Warning Signals of imminent phase transitions. A publicly available multivariate time series is analyzed using so-called cumulative Recurrence Networks (cRN), which are recurrence networks with edges weighted by recurrence time and directed towards previously observed data points. The results are compared to previous analyses of the same data set, benefits, limitations and future directions of the analysis approach are discussed.

## Introduction

The detection of Early Warning Signals (EWS) of imminent transitions in states of the body and mind has been a topic of great interest in the sciences that study health and wellbeing. In a clinical setting, such transitions often concern sudden changes in symptom severity associated with the onset of disease or psychopathology (see e.g., Leemput et al., 2014; Cramer et al., 2016; Fartacek et al., 2016; Wichers et al., 2016), or, conversely, they might herald onset of recovery due to psychological or medical interventions (see e.g., Kowalik et al., 2010; Lichtwarck-Aschoff et al., 2012; Olthof et al., 2019a, Olthof et al., 2019b). These transitions are often conceived of as the phase transitions of complex dynamical systems (cf. Olthof et al., 2022) and the EWS generally refer to well-known critical phenomena observed around the tipping points of thermodynamical, chemical and ecological systems (Scholz et al., 1987; Scheffer et al., 2009; Lenton, 2013).

Most EWS are so-called indicators of a loss of resilience (Scheffer et al., 2018), that is, a state of the system that is currently stable starts to display characteristic signs of instability such as critical slowing down, or, critical fluctuations. Critical slowing down refers to the increase in return times after perturbation which can be directly observed in perturbation experiments (Scholz et al., 1987), or, can be inferred from data, for example by evidencing an increase in magnitude of short-range temporal autocorrelations or variance (Leemput et al., 2014; Weinans et al., 2021). Critical fluctuations can be evidenced by a change in power law scaling behavior (e.g., Bak et al., 1987; Stephen et al., 2009), an increase in fluctuation intensity (e.g., Kelso et al., 1986; Fartacek et al., 2016; Olthof et al., 2019b) or an increase of entropy and related measures (e.g., Stephen et al., 2009; Lichtwarck-Aschoff et al., 2012). The ability to detect such periods of destabilization is hypothesized to be informative for the optimal planning of interventions which attempt to facilitate a transition to a more healthy state. There is also a great potential use in the context of prevention, that is, EWS could be used for the timing of activities intended to prevent a maladaptive state to emerge in the first place (e.g., Schiepek et al., 2011; Schiepek et al., 2016; Olthof et al., 2022). It is important to note that EWS like critical fluctuations and critical slowing down are associated with particular types of transitions that involve one or more control parameters of the system to approach a critical value. Not all order transitions can be attributed to changes in control parameters (Burthe et al., 2016; Guttal et al., 2016), an apparent transition to a qualitatively different mode of behavior can for example be noise-induced, continuous, or, due to nonlinear dynamics evolving within a specific regime without a change in control parameters (see e.g., Lenton, 2013). For the purpose of the present paper we will assume the data under scrutiny contain at least one critical transition that is expected to be preceded by EWS.

### Weak Versus Strong Complexity Assumptions

The multivariate time series data analyzed in the literature on health and wellbeing mostly concern self-reports of human experience collected through the Experience Sampling Method (ESM), or Ecological Momentary Assessment (EMA) in which participants are prompted one or more times per day to answer some questions, which are often self-ratings of psychological or physical states. Another data source concerns the measurements of physiological variables recorded by wearable sensors. Both types of data are known to be non-stationary, contain sudden shifts in level, trend, or variance (Shiffman, 2014; Nezlek, 2015; Schiepek et al., 2016) and contain evidence of long-range temporal correlations and power-law scaling (Delignières et al., 2004; Hasselman and Bosman, 2020; Olthof et al., 2020).

Many researchers do acknowledge the data generating processes underlying time series appear to violate the ergodic assumptions of stationarity, homogeneity (Molenaar, 2004) and memorylessness (Ramachandran, 1979), but will consider these facts to be nuisance factors that should be removed from the data, or handled by (statistical) models as a covariates or random effects (*weak complexity assumption*).

One approach suggested to “deal with” non-stationarity is to collect data during a short, but very intensive, measurement period, for example, one or 2 weeks in which a participant is prompted 10 or more times per day to provide self-reports of activities, emotional, psychological or physiological states (Trull and Ebner-Priemer, 2009; Smith, 2012; Liao et al., 2016). A post-data collection approach is data-segmentation based on the identification of relatively stable or homogeneous epochs in the time series data and fit a model for each stable epoch separately (Bak et al., 2016; Wichers et al., 2016). This is of course disrupting potential long range dependencies in the data and introduces a problem for use in practice: The epochs can only be identified and analyzed after the data collection has ended. Less destructive, but still aimed at removing potentially nonlinear phenomena are the practices of detrending, “artefact” removal and imputation (Adolf and Fried, 2019; Piccirillo and Rodebaugh, 2019). On the data analysis side, these phenomena are often modeled as nuisance factors, noise, or, explicitly as model parameters, for example, time varying parameters to deal with nonstationarity like Time Varying Auto Regressive models (TV-AR, Bringmann et al., 2017), identifying homogeneous subgroups to deal with heterogeneity (GIMME, Beltz and Gates, 2017; Gates et al., 2014), or, using fractional integration components to deal with long-range dependence (ARfiMA, Diniz et al., 2011; Torre and Wagenmakers, 2009; Hasselman, 2013). These statistical models generally limit estimating temporal dynamics to the linear domain and very short time scales (e.g., lag-1 vector autoregression Epskamp et al., 2012). This is also the case for network models estimated from time series, like the Gaussian Graphical Model (GGM, Epskamp et al., 2012) and the Ising graphical model (Van Borkulo et al., 2014). Finally, a practice that is commonly observed is the aggregation of time series, which often concerns the simple averaging of different time series to achieve dimension reduction. In general, such naive approaches to the aggregation of time series are not recommended (cf., Petitjean et al., 2011). As a consequence, the techniques used under the weak complexity assumption greatly reduce the range of potential data generating processes that can be considered to underlie the observed (multivariate) time series.

These examples also reveal that current methods (measurement and data analysis) and models (inference and interpretation) used to detect EWS in time series data of physiological and psychological variables impair their potential for being applicable in a clinical setting. The first, rather obvious point is that clinical practice requires personalized, idiographic methods, that is, EWS should be reliably detectable in data observed in a single individual and ideally make use of the particular facts pertaining to the case (personal history, social-economic context, pre-existing conditions, etc.). This requirement excludes all methods that have been used in studies to evidence EWS based on samples of many individuals (e.g., Leemput et al., 2014). Second, although methods and models from complexity science are available, many authors focus on developing (linear) statistical models to evidence EWS. Two recent reviews of the personalized approach to psychopathology (Piccirillo and Rodebaugh, 2019; Wright and Woods, 2020) fail to discuss, or even mention, the use of complexity methods. As a consequence analyses often have to be conducted post-data collection, which renders the techniques impractical for analyzing and intervening on continuously sampled patient data. Studying which EWS predicted a transition, after the transition has already occurred may be of value to the researcher, but the clinician has to be able to make decisions in the here and now.

#### The present study: “First Analyze, then Aggregate!”

It is the purpose of the present paper to showcase an analytic approach to detecting EWS under a *strong complexity assumption*, that is, analytic techniques will be used that were developed to quantify the dynamics of complex systems, even in the context of nonstationary and nonhomogeneous time series data. Moreover, the explicit goal is to provide an analysis strategy that meets the requirements for potential use as a tool in a clinical setting for the diagnosis and treatment of individuals. The analysis strategy is based on recurrence networks, to which some constraints are added, resulting in a so-called *cumulative Recurrence Network* (cRN). The cRN is constructed from a recurrence matrix based on measurements of the state variables of the system (Marwan et al., 2007; Wallot et al., 2016) and is a directed, weighted, network (Zou et al., 2019) in which the nodes represent time points, the edges connect recurring values and the weights represent the distance (in time) between two recurring values. The direction of the edges is always towards previously observed time points (out-degree only). This mimics the situation in which only data up to the most recently observed point in time is available to decide on the presence of EWS. In order to show we can dispense with unnecessary aggregation or dimension reduction of multivariate time series data, cRN representing the phase spaces of different subsystems will be constructed into a Multiplex Recurrence Network (Zou et al., 2019).

**FIGURE 1:**
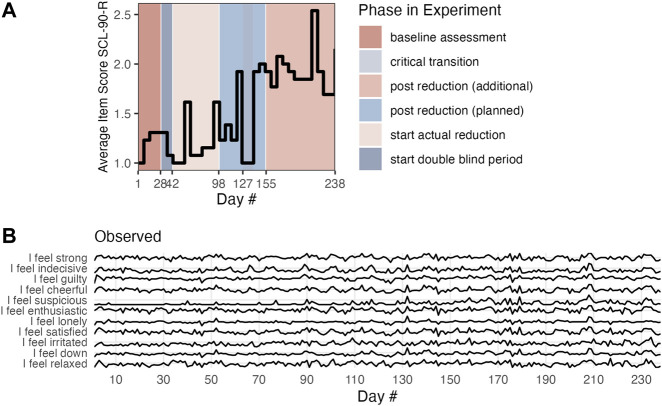
Panel **(A)** shows the state variable (SCL-90-R). Panel **(B)** is an example of the subset of Mood time series.

To demonstrate the potential of Multiplex cumulative Recurrence Network analysis for multivariate time series data, a publicly available data set was analyzed (the data were published as: Kossakowski et al., 2017). The data were acquired in the context of a double-blind N = 1 experiment in which the antidepressant medication of a participant diagnosed with Major Depressive Disorder (MDD) was gradually reduced (Wichers et al., 2016). The participant generated 1,478 questionnaire responses (on average 43.4 per day) over a period of 239 consecutive days. The purpose of the original paper was to detect *critical slowing down* as an EWS for the critical transition that occurred. The transition concerned going from a non-depressed state to a depressed state, likely due to the reduction of antidepressant medication. Figure 1 panel A, displays the mean of weekly measurements of the SCL-90-R depression scale, which will be interpreted as the global state variable in which the transition is observable. During the time of the critical transition, data collection was partly stopped, so the apparent drop in symptom severity in fact reflects a period in which the global state variable was not observed. The daily measurements will be analyzed for the presence of EWS. It is assumed the multivariate data at least approximately capture the internal state dynamics of several different subsystems of the entire phase space that represents the micro-scale configurations from which the macroscopic, system-wide depressive state emerges.

First, a brief overview of recurrence analysis and recurrence networks is provided, second, the concept of the (multiplex) cRN is introduced as a tool for analyzing multivariate time-series data obtained through the real-time monitoring of an individual. Finally the technique is applied to the data and compared to the original analyses by Wichers et al. (2016).

## Time Series Analyses Based on Recurrences in Phase Space

Recurrence Quantification Analysis and its derivatives (Marwan et al., 2007; Cox et al., 2016) are not new to the behavioral and life sciences and have been successfully applied to study the temporal dynamics underlying human physiology (Schinkel et al., 2009; De Graag et al., 2012), motor coordination (Shockley et al., 2003; Aßmann et al., 2007; Wijnants et al., 2011), perception (Hasselman, 2015), language (Wijnants et al., 2012; Wallot et al., 2013), cognitive performance (Stephen et al., 2009; Oomens et al., 2015) and dyadic interaction (Dale and Spivey, 2006; Louwerse et al., 2012). A large number of studies use RQA to quantify dynamics of physiological or performance measurements in different populations, for example comparing literacy skills in average and dyslexic readers (Wijnants et al., 2012; Hasselman, 2015), or searching for biomarkers of Asperger’s syndrome, schizophrenia, ASD (Fusaroli et al., 2013, 2016), absence epilepsy (Bosl et al., 2017) and speech pathology (Little et al., 2007).

Recurrence analytic approaches to time series analysis are essentially model-free (descriptive) and make only a few assumptions about the data related the observation of dynamics/variance (Bianciardi et al., 2007). Moreover they allow for:

• The description of linear as well as nonlinear dynamical phenomena (Marwan et al., 2007).
• The description of (transitions between) dynamical regimes, even in exceptionally noisy environments (Zbilut et al., 1998).
• The quantification of recurrent dynamics across all available time scales (Marwan et al., 2007; Donner et al., 2011).
• The quantification of attractor geometry across all available time scales (Zou et al., 2012).
• The quantification of structural similarities between different time series represented as Multiplex Recurrence Networks (Zou et al., 2019), suspending the need for potentially problematic aggregation and/or dimension reduction of multivariate time series data.

It is not the case that results from recurrence-based analyses are immune, or “robust” to nonstationarity and nonhomogeneity, rather, it is the case that these methods can be understood as descriptive of such dynamics. The goal is not to infer a true model, process or estimate a population model parameter, but to characterize the dynamics displayed during the observation time, either as observed, or, after a reconstruction of the phase space.

### Recurrence Quantification Analysis

Recurrence analyses are based on a recurrence matrix which represents the states of the system that are re-visited at least once during the time the system was observed. If a sequence of states is recurring, this is referred to as a trajectory in phase space, a relatively stable state, or, orbit of the system. In this paper the observed variables in a multivariate time series are considered to be measurements of the state variables of the system (cf. Marwan et al., 2007; Wallot et al., 2016).

An observed time series can be interpreted as a finite representation of the trajectory or state-evolution of a stochastic or deterministic dynamic system: ${y_{i}}_{i=1}^{N}$ , with *y*_*i*_ = *y* (*t*_*i*_) (cf. Zou et al., 2019). The open data set analysed in the present paper is a multivariate time series of at least 70 variables observed in a single participant over the period of 239 days. The variables can be conceptually grouped into six subsystems (*Mood*, *Physical*, *Self Esteem*, *Mental Unrest*, *Sleep* and *Day*). Each time series is considered a state vector $\vec{y_{i}}$ of the *m*-dimensional phase space of the (sub)system. For example, the Mood subsystem consists of 12 time series representing a 12-dimensional state space. The 12 values that were simultaneously observed at each time point represent a specific Mood-state, which can be regarded as a 12-tuple coordinate in the 12 dimensional space. As the observed values change over time, they trace a trajectory through this space representing the mood dynamics of the participant. By evaluating the observed values as coordinates it is possible to quantify whether the system (approximately) returns to the same regions and revisits previously traversed trajectories. In the current context this would refer to recurring emotional states.

**FIGURE 2:**
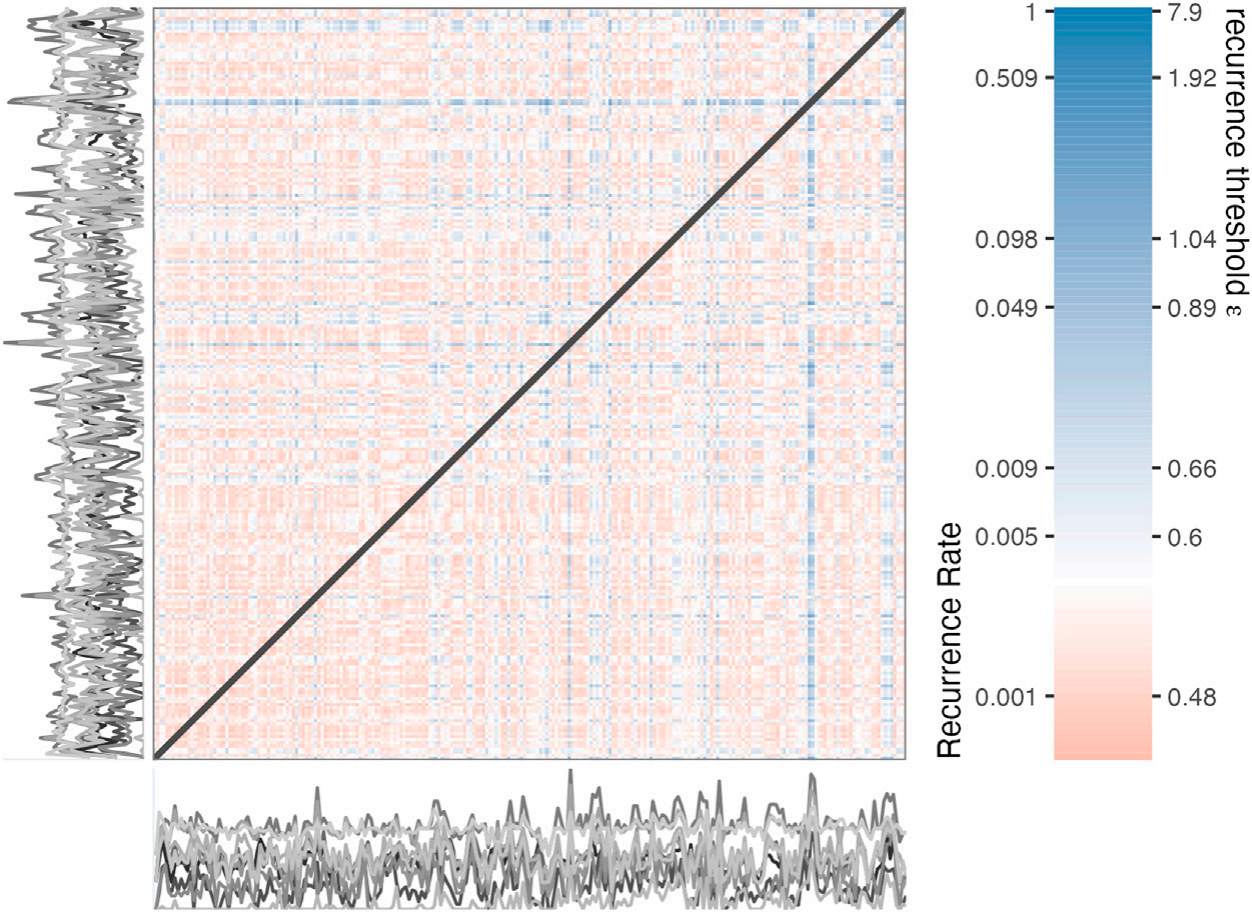
Distance matrix based on 12 time series (displayed as grey lines) representing self-reports of the mood of an individual.

The recurrence matrix **R**_*i*,*j*_ is defined as:

$$R_{i,j}\left(\varepsilon \right)=\Theta \left(\varepsilon -\|{\vec{y}}_{i}-{\vec{y}}_{j}\|\right),\;\;\;i,\;j=1,\ldots ,N$$ (1)

where ‖ ⋅ ‖ is a distance norm (e.g., Euclidean, Chebyshev, Manhattan) calculated for each coordinate relative to every other coordinate, *ɛ* is a threshold value for the distances between coordinates, and Θ is the Heaviside function, which returns one if a distance value falls below *ɛ* and 0 otherwise. The threshold value *ɛ* directly determines how many recurring values appear in the matrix, which is called the Recurrence Rate (proportion of recurrent points in **R**_*i*,*j*_). Figure 2 represents the distance matrix of all observed coordinates in the 12-dimensional Mood phase space. The color-bar displays distance values on the right side, which, should they be chosen as the threshold value *ɛ*, would result in the Recurrence Rate displayed on the left.

Many other measures can be calculated from the matrix, such as the proportion of recurrent points that form line structures which represent larger patterns of recurring values (Marwan et al., 2007; Donner et al., 2011).

### Recurrence Networks

**FIGURE 3:**
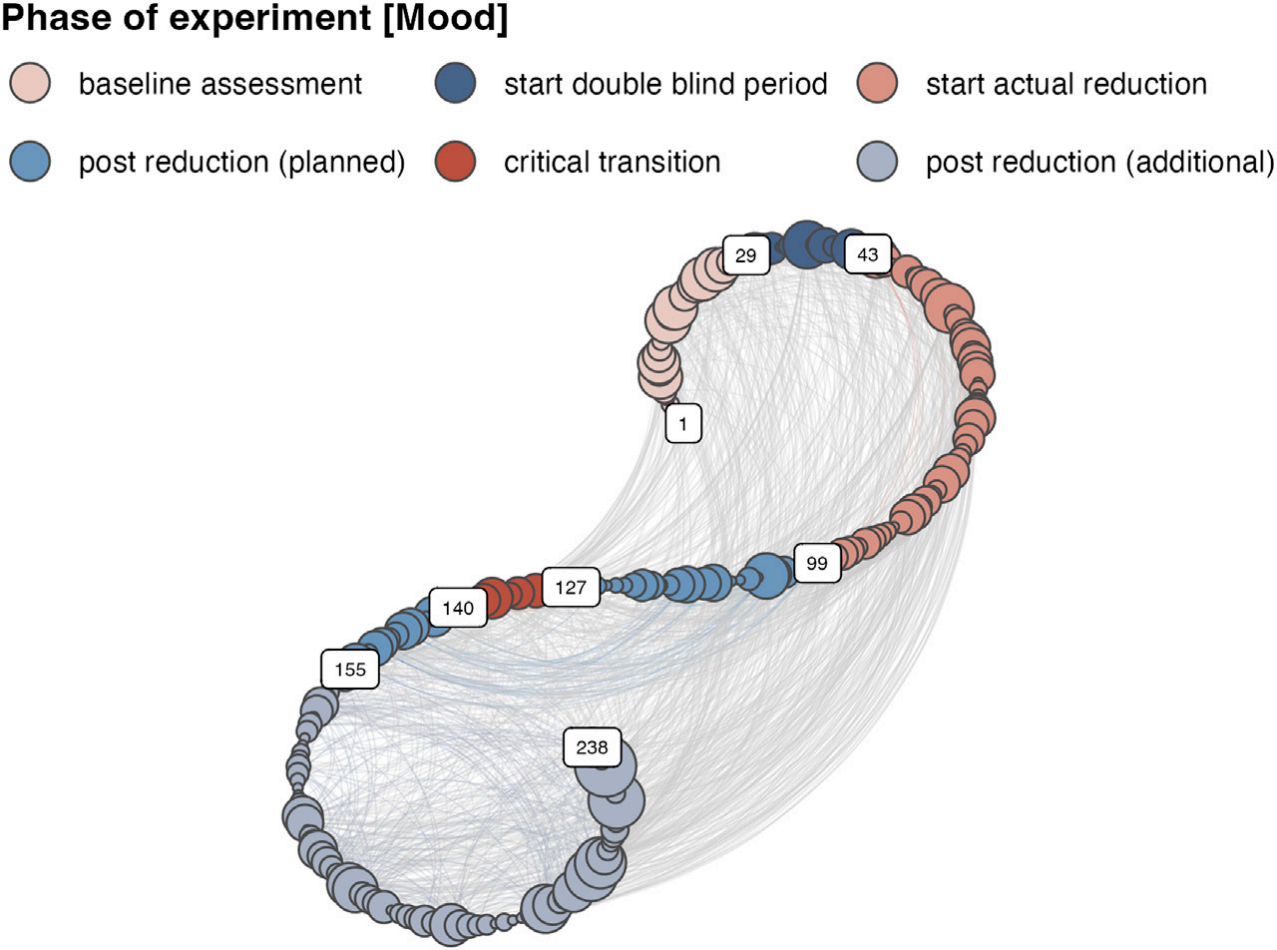
A Recurrence Network in spiral layout based on the distance matrix of 12 time series representing self-reports of the mood of an individual recorded over a period of 238 days.

To create a recurrence network, we consider the recurrence matrix **R**(*ɛ*) to be an adjacency matrix **A**(*ɛ*) of an adjoint complex network. By removing the diagonal this matrix represents an unweighted, undirected, simple graph called an *ɛ*-Recurrence Network (Marwan et al., 2007; Donner et al., 2011). The vertices *V*_*i*_ of the network represent the state coordinates and are indexed by their time order. The edges indicate whether the state observed at one point in time will recur at another point in time and this is of course conditional on the threshold *ɛ*. It is also possible to create a weighted network, either by keeping the distances that are smaller than *ɛ*, or, by creating edge weights that are based on the time between recurring states, the *recurrence time*, or *recurrence time frequency*. (Hasselman and Bosman, 2020). Figure 3 displays the *ɛ*-RN based on the distance matrix shown in Figure 2 with a threshold of *ɛ* of 0.889 yielding a Recurrence Rate of 0.05. The RN is presented in a so-called spiral layout, which preserves the time order of the vertices (see Hasselman and Bosman, 2020).

Recurrence networks are formally equivalent to Random Geometric Graphs (RGG), a full analytic theory of *ɛ*-RN’s is provided by Zou et al. (2019). For the purpose of the present paper it is important to understand the differences between the information obtained about the phase space trajectory based on RQA measures and the complex network measures extracted from the *ɛ*-RN. The line structures analyzed in RQA represent the evolution of state coordinates, so those measures quantify dynamic properties of the trajectory in phase space. Most network measures extracted from a *ɛ*-RN will quantify topological or geometric properties of the attractor (Zou et al., 2019), when used as in the context of EWS, they can be referred to as geometric resilience loss indicators. In a *ɛ*-RN local and global measures can be identified for roughly two types of measure: Path- and Neighborhood-based measures. For the present study, we focus on Neighborhood-based measures.

In what follows a description of unweighted, undirected local and global characteristics of the vertices in a Recurrence Network is provided. This description is not exhaustive, but covers the most important vertex characteristics for the present context (descriptions and formula’s presented here were adapted from various sources, most can be found in Zou et al., 2019).

### Local Complexity Measures

Graph-neighborhood measures calculated from a *ɛ*-RN capture information about the local density or space filling behavior of the phase space trajectory. This has led to the novel measures such as the notion of a transitivity dimensions and local clustering dimensions (Donges et al., 2012; Zou et al., 2019), which evaluate the dependence of these measures on the threshold value *ɛ*. In the present context more familiar network measures will be presented, starting with the *degree centrality* of a vertex ${\hat{k}}_{i}\left(\varepsilon \right)$ :

$${\hat{k}}_{i}\left(\varepsilon \right)=\overset{N}{\underset{i,\;j=1}{\sum }}A_{ij}\left(\varepsilon \right),$$ (2)

Where *A*_*ij*_(*ɛ*) is the Adjacency matrix. In *ɛ*-RNs the local degree is normalized to the time series length, rather than to a theoretical maximum, and the *degree density* is calculated as:

$${\hat{\rho }}_{i}\left(\varepsilon \right)=\frac{{\hat{k}}_{i}\left(\varepsilon \right)}{N-1}$$ (3)

The degree density of a vertex *V*_*i*_ indicates the probability that a randomly chosen state represented by *V*_*j*_ is *ɛ*-close to the state represented by *V*_*i*_. It can be interpreted as a localized recurrence rate.

Another value is the *local clustering coefficient* ${\hat{C}}_{i}\left(\varepsilon \right)$ , which measures the fraction of pairs of vertices around *V*_*i*_ that are *ɛ*-close:

$${\hat{C}}_{i}\left(\varepsilon \right)=\frac{1}{{\hat{k}}_{i}\left({\hat{k}}_{i}-1\right)}\overset{N}{\underset{j,\;h=1}{\sum }}A_{ij}A_{ih}A_{jh}$$ (4)

This measure quantifies the geometric alignment of the state vectors ${\vec{y}}_{i}$ , which occurs for example close to dynamic invariant structures in phase space like quasi-periodic behavior or unstable periodic orbits (UPOs, Zou et al., 2019).

The degree density and clustering coefficient quantify vertex properties at a local scale, a neighborhood, whereas measures based on shortest paths quantify vertex connectivity relative to the scale of the network as a whole. The *closeness centrality* measure ${\hat{c}}_{i}\left(\varepsilon \right)$ is defined as the inverse of the arithmetic mean of shortest path lengths *l*_*ij*_:

$${\hat{c}}_{i}\left(\varepsilon \right)={\left(\frac{1}{N-1}\overset{N}{\underset{j=1}{\sum }}l_{ij}\right)}^{-1}$$ (5)

The *local efficiency* is the inverse geometric mean of *l*_*ij*_:

$${\hat{e}}_{i}\left(\varepsilon \right)=\frac{1}{N-1}\overset{N}{\underset{j=1}{\sum }}l_{ij}^{-1}$$ (6)

In the path length calculation ‘disconnected’ vertices are assigned a local average path length of *N* − 1. This occurs quite often in *ɛ*-RN, a state occurring at *V*_*i*_ does not recur at every other vertex by construction of the matrix **R**(*ɛ*). It is known that the efficiency measure ${\hat{e}}_{i}\left(\varepsilon \right)$ is well-behaved, even if the graph has many disconnected components (cf. Boccaletti et al., 2006)

### Global Complexity Measures

Table 1 displays some of the global measures commonly used to describe the structure of complex networks. The *edge density* $\hat{\rho }\left(\varepsilon \right)$ is simply the average of the local degree density. The *global clustering coefficient* $\hat{C}\left(\varepsilon \right)$ is the arithmetic mean of the local clustering coefficients (cf. Watts and Strogatz, 1998). An alternative definition exists called *network transitivity* $\hat{T}\left(\varepsilon \right)$ , which represents the effective global dimensionality of the system (Barrat and Weigt, 2000) as opposed to the average local dimensionality in phase space quantified by $\hat{C}\left(\varepsilon \right)$ . The *average path length* $\hat{L}\left(\varepsilon \right)$ and *global efficiency* $\hat{E}\left(\varepsilon \right)$ are the arithmetic mean of the inverse local closeness, and the inverse arithmetic mean of the local efficiency, respectively (see Zou et al., 2019 for details). The *edge density* is equal to the *Recurrence Rate* in RQA.

**TABLE 1:** Global recurrence network measures.

	Mood	Physical	Self Esteem	Mental Unrest	Sleep	Day
Recurrence Threshold *ɛ*	0.889	0.389	0.283	0.443	1.237	1.331
Edge Density $\hat{\rho }\left(\varepsilon \right)$	1.662	1.791	2.323	1.753	2.052	6.524
Global Clustering $\hat{C}\left(\varepsilon \right)$	0.17	0.18	0.22	0.19	0.17	0.27
Network Transitivity $\hat{T}\left(\varepsilon \right)$	0.49	0.48	0.58	0.55	0.49	0.63
Average Path Length $\hat{L}\left(\varepsilon \right)$	74.2	60.6	42.1	61.0	103.5	11.0
Global Efficiency $\hat{E}\left(\varepsilon \right)$	245.857	752.248	484.290	353.830	1,577.394	92.836

### Cumulative Recurrence Networks for Real-Time Process Monitoring

**FIGURE 4:**
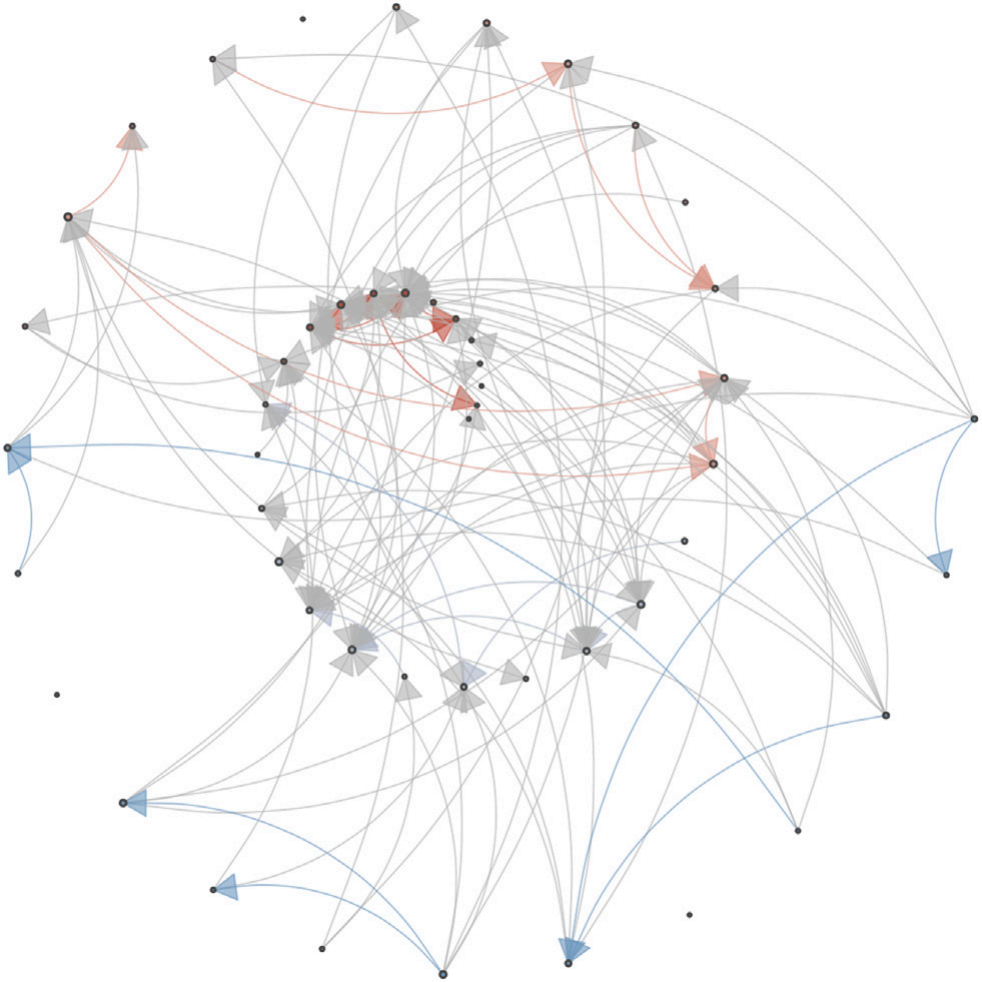
The first 50 vertices of a weighted, directed cumulative Recurrence Network (cRN) based on the lower triangle of the recurrence matrix of the Mood subsystem.

In order to fulfill the goal of serving as a potential tool for assessing an imminent regime change in a clinical, real-time monitoring setting, we should not use any information about states that recur at some point in the future, because these are of course only known ex post facto. It is possible to use windowed analyses on the time series and construct a recurrence matrix based on a right aligned window and compute RQA or *ɛ*-RN measures within each of the sliding windows. The minimal window size is of course limited by the time series length, but also cannot be too small. To achieve the same goal, it is also possible to create a directed (weighted) network with recurrent points based on an adjacency matrix with the upper triangle set to zero. The structure of the network is evaluated based on measures that only consider the vertex out-degree. Such a network represents vertex properties that are cumulative with respect to the number of edges that represent whether the current vertex is a recurring state of the past, a cumulative Recurrence Network (cRN). Figure 4 displays the first 50 vertices of a directed, weighted network, with edges connecting only to vertices of a lower time index.

### Multiplex (Cumulative) Recurrence Networks

**FIGURE 5:**
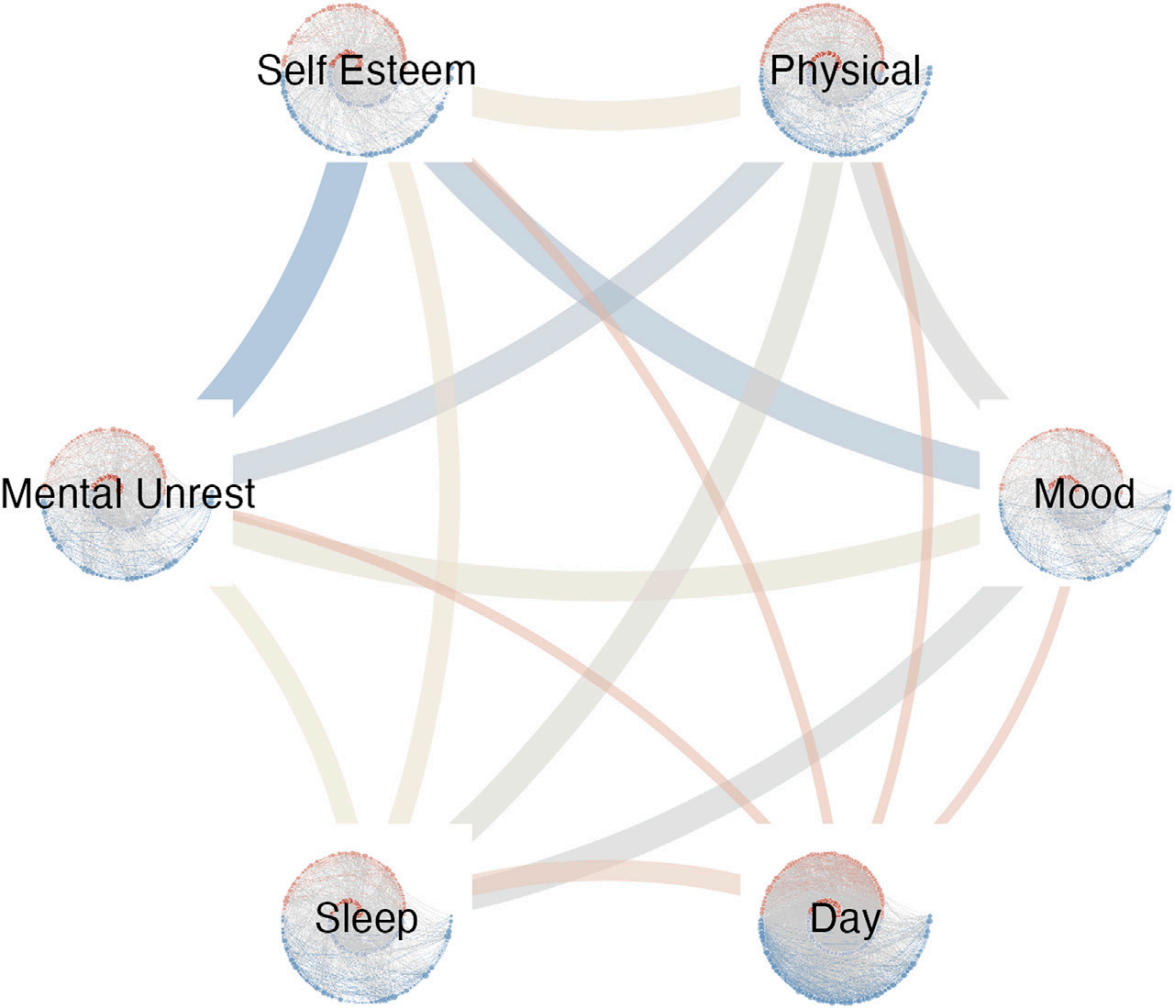
The Multiplex network with each cRN as a layer. The edge weights and color represent the magnitude Inter-layer Mutual Information.

Multiplex recurrence networks provide a framework for studying the temporal structure in multivariate records of the observables of a complex dynamical system. Multiplex networks have recently been constructed from horizontal visibility graphs (HVG, Lacasa et al., 2015) and recurrence networks (Eroglu et al., 2018). To obtain a *Multiplex Recurrence Network* (MRN), the *M* time series of length *n* in the multivariate data set are turned into *M* recurrence networks, each with *n* nodes, to constitute the *κ* = *M* layers of he MRN. In the present example the MRN is built from six weighted, directed recurrence networks, that represent cumulative time by only considering the vertex out-degree when computing vertex properties, see Figure 5

### Multiplex Recurrence Network Measures

The purpose of constructing a multi-layer network is to evaluate the structural similarities between the dimensions of the multivariate time series that constitute the layers. Formally, based on the *M*-dimensional time series ${\left[{\vec{X}}_{t}\right]}_{t=1}^{N}$ with ${\vec{X}}_{t}=\left.\left(x_{t}^{\left[1\right]},x_{t}^{\left[2\right]},\ldots ,x_{t}^{\left[M\right]}\right)\right)$ , RNs can be constructed for each of the *α* components of ${\vec{X}}_{t}$ (cf. Zou et al., 2019). The multiplex network can be represented as a giant *NM* × *NM* matrix A , with a diagonal of *M* adjacency matrices *A* ^[*α*]^ and off-diagonal identity matrices *I*_*N*_ of size *N* × *N*:

$$A=\left[\begin{array}{cccc} A^{\left[1\right]} & I_{N} & \ldots & I_{N}\\ I_{N} & A^{\left[2\right]} & \ddots & \vdots \\ \vdots & \ddots & \ddots & I_{N}\\ I_{N} & \ldots & I_{N} & A^{\left[M\right]} \end{array}\right]$$ (7)

In practice, the giant matrix is not constructed, rather, a projection of A as a weighted matrix with the RN as vertices connected by edges. The association between layers *α* and *β*, (*I*_*α*,*β*_) in the MRN can be quantified by some measure of association, for example, the *Inter-layer Mutual Information*, *Inter-layer Correlation*, which are based on the degree distributions $P\left(\kappa^{\left[\alpha \right]}\right),P\left(\kappa^{\left[\beta \right]}\right)$ (Lacasa et al., 2015; Eroglu et al., 2018). The mutual information is calculated as:

$$I_{\alpha ,\beta }=\underset{\kappa^{\left[\alpha \right]}}{\sum }\underset{\kappa^{\left[\beta \right]}}{\sum }P\left(\kappa^{\left[\alpha \right]},\kappa^{\left[\beta \right]}\right)\log\frac{P\left(\kappa^{\left[\alpha \right]},\kappa^{\left[\beta \right]}\right)}{P\left(\kappa^{\left[\alpha \right]}\right),P\left(\kappa^{\left[\beta \right]}\right)}$$ (8)

The term $P\left(\kappa^{\left[\alpha \right]},\kappa^{\left[\beta \right]}\right)$ denotes the joint probability of observing the same node with degree *κ* ^[*α*]^ in layer *α* and degree *κ* ^[*β*]^ in layer *β*. $P\left(\kappa^{\left[\alpha \right]}\right)$ and $P\left(\kappa^{\left[\beta \right]}\right)$ represent the marginal probabilities of observing the degree distribution in each layer. Taking *I*_*α*,*β*_ as the weight between two layers in a multiplex network can be interpreted as the quantification of structural regularities between the time series in the multivariate data set. Instead of the mutual information, one can simply calculate the Pearson correlation between the degree vectors *R*_*α*,*β*_. The averaged mutual information $\left\langle I_{\alpha ,\beta }\right\rangle$ or correlation $\left\langle R_{\alpha ,\beta }\right\rangle$ across all layers in a multiplex network can be interpreted as a quantification of typical information flow between the dimensions of the multivariate system (Lacasa et al., 2015; Eroglu et al., 2018). In the present application, the *I*_*α*,*β*_ between individual layers indicates the characteristic way in which self-reports of internal states are structurally associated across all observed timescales in the multivariate dataset. If the layers of the MRN are weighted RN (denoted as MwRN, or McRN), the Multi-layer MI and correlation are based on the strength distributions in each layer and can be constructed as the association between $P\left(s{\left(\kappa \right)}^{\left[\alpha \right]}\right)$ and $P\left(s{\left(\kappa \right)}^{\left[\beta \right]}\right)$ (cf. Hasselman and Bosman, 2020):

$$I_{\alpha ,\beta }=\underset{s{\left(\kappa \right)}^{\left[\alpha \right]}}{\sum }\underset{s{\left(\kappa \right)}^{\left[\beta \right]}}{\sum }P\left(s{\left(\kappa \right)}^{\left[\alpha \right]},s{\left(\kappa \right)}^{\left[\beta \right]}\right)\log\frac{P\left(s{\left(\kappa \right)}^{\left[\alpha \right]},s{\left(\kappa \right)}^{\left[\beta \right]}\right)}{P\left(s{\left(\kappa \right)}^{\left[\alpha \right]}\right),P\left(s{\left(\kappa \right)}^{\left[\beta \right]}\right)}$$ (9)

Another measure that can be used to represent the weights in the MRN is the *Edge Overlap* *ω*. It represents the proportion of edges that are shared between any two vertices across all layer-pairs in the multiplex network (Eroglu et al., 2018):

$$\omega =\frac{\sum_{i}\sum_{j>i}\sum_{\alpha }A_{ij}^{\left[\alpha \right]}}{M\sum_{i}\sum_{j>i}\left(1-\delta_{0,\sum_{\alpha }A_{ij}^{\left[\alpha \right]}}\right)},$$ (10)

Where *δ*_*ij*_ is the Kronecker delta. The edge overlap measure estimates similarity and coherence between network layers and its average $\left\langle \omega \right\rangle$ , is a global network measure of coherence between the different layers which, together with the averaged Inter-layer MI, can be used to detect transitions between dynamic regimes in the multivariate time series (see e.g., Eroglu et al., 2018).

Once the MRN is constructed, it is possible to calculate any of the aforementioned network measures. The local measures now provide information about the properties of the layer relative to the other layers in the MRN. In order to get a time course of these layer association measures a windowed analysis can be conducted on the MRN.

The MRN allows for the study of structural similarities between the network layers. In the present context this refers to subsets of the different ESM variables in the multivariate data set, which were considered the dimensions of a subsystem phase space. The MRN structure is expected to change near tipping points due to the reconfiguration of the available degrees of freedom in phase space and the creation of new associations between different dimensions of the phase space. Note that all the time series are in principle still accessible, that is, there was no aggregation of observations. For the MRN, it is possible to evaluate which layers might be responsible for structural changes, but it is also possible to evaluate the role of individual variables within the RN by evaluating the contribution of each dimension to the recurring phases of the system (see Heino et al., 2021 for an example).

### Analysis Strategy

Cramer et al. (2016) used a window of 30 days to calculate the autocorrelation function and variance as an EWS, which were both expected to increase around the critical transition. The windowed function was calculated from a detrended, overall mental state variable, which the authors describe as “the total mental state score based on a moving window over time”. For data analysis the authors present a Gaussian Graphical Model (GGM) fitted to different epochs in the experiment, prior to the transition. The GGM consisted of five nodes, three of which represented different aspects of the mental state variable consisting of sets of time series selected from the multivariate data set. The time series were detrended and aggregated to yield a mean series representing: *positive affect* (“content”, “cheerful”, “enthusiastic”); *mental unrest* (“restless”, “agitated”, “indecisive”); *negative affect* (‘irritated”, “lonely”, “anxious”, “guilty”). The other two nodes consisted of a single observed time series *worry* and *suspicious*. It is likely the authors chose these specific variables from the set of about 70 available variables based on theoretical and practical considerations, but they do not explain why and how the time series were aggregated and subsequently analyzed in a moving window. Time series aggregation should be done with care, especially if the series are correlated (Wigley et al., 1984) or otherwise temporally aligned (Petitjean et al., 2011).

Irrespective of the validity of time series aggregation, or the specifics of the method used, the effect of time series aggregation is data reduction, and data smoothing. The use of moving windows is warranted because the aim is to detect changes in the correlational structure and variance of the time series, however, sliding window statistics will introduce smoothing in addition to the averaging of the time series. Finally, detrending is a basic method for dealing with non-stationary time series, but it does not guarantee a stationary series if there are multiple stable levels, nonlinear trends, discontinuous changes or heterogeneity of variance. Such phenomena will almost certainly occur in ESM data due to the bounded nature of self-ratings on a an ordinal scale combined with the long observation time: The projection function of the internal state onto the ordinal scale will likely not be constant over 200 + days. Olthof et al. (2020) also analyzed the Cramer et al. (2016) data set and indeed found evidence for long-range dependence, non-stationarity of the autocorrelation function and divergence (nonlinear prediction error) in many of the time series in the data set.

The first analysis will try establish whether the major transition in the state variable can indeed be considered a critical transition. To do so, the data-segmentation approach from the original paper will be followed, by creating McRN for each of the phases of the experiment. The difference being that the McRN will include all relevant variables This method does not meet the goals set in the introduction, as it can only be performed after the entire series has been observed. It does provide an opportunity to compare the two methods. The second analysis will concern a windowed analysis of the time evolution of the different measures that quantify structural similarities between the layers of the multiplex network and evaluate whether they could serve as Geometric Resilience Loss Indicators.

### Data Preparation

The original data set contains over 80 variables, several of which concern date and time information, or, concerned answers to questions that were only triggered in certain contexts. There were 42 variables containing densely sampled self-ratings, for the analyses in this paper 32 variables were used. Selection of variables was based on a support criterion: Only variables with less than 5% missing values were considered and in addition, the differenced series had to contain less than 33% zeroes (indicative of sufficient dynamics) to be included in the analysis. An exception was made for a set of variables that contained more than 5% missing due to the fact they were prompted only in the morning (about the quality of sleep the previous night) and in the evening (about the experience of the day). These variables were included because of their theoretical relevance (e.g., their potential dual role as symptom and cause (cf. Cramer et al., 2016)) and because the aggregation level was at the level of a day which is what the questions assessed (e.g., “I found this a nice day” rated in the evening). The 32 variables were grouped into subsets that can conceptually be considered to be subsystems: *Mood*, *Physical*, *Self Esteem*, *Mental Unrest*, *Sleep* and *Today*. Because some of the variables of interest were observed several times each day, while others only less frequently or only once per day each variable was aggregated so the sampling frequency was 1/day. The rating scales that were used could also vary between variables, therefore, all variables were rescaled to a range of one–7. Some variables were also recoded, for example, the questions asking to rate the experience of a positive mood (e.g., “I feel strong”) were reversed such that higher ratings represent a less positive mood. Any missing values were imputed using multivariate imputation by chained equations based on each subset of variables (using R package *mice*, Buuren and Groothuis-Oudshoorn, 2011). Table 2 lists which variables were grouped with each subset.

**TABLE 2:** Variables used to construct Recurrence Networks.

Subset	Variable name
Mood	mood_relaxed^a^, mood_down, mood_irritat, mood_satisfi^a^, mood_lonely, mood_enthus^a^
	mood_suspic, mood_cheerf^a^, mood_guilty, mood_doubt, mood_strong^a^
Physical	phy_hungry, phy_pain, phy_headache, phy_physact^a^
SelfEsteem	se_selflike^a^, se_selfdoub, se_handle^a^
Patience	pat_restl, pat_agitate, pat_worry, pat_concent^a^
Sleep	mor_asleep, mor_nrwakeup, mor_lieawake, mor_qualsleep^a^, phy_tired, phy_sleepy
Day	evn_ordinary, evn_niceday^a^, evn_inflmood, mor_feellike^a^

a Variable was recoded.

The output and figures displayed in this paper were created with R-package *casnet* (v. 0.2.1, Hasselman, 2022), there are however many more ways to conduct the recurrence-based analyses discussed here (e.g. in Python, Matlab, or stand-alone software). A comparison of software packages for performing RQA can be found here: https://github.com/JuliaDynamics/RecurrenceAnalysis.jl/wiki/Comparison-of-software-packages-for-RQA.

## Results

### Comparison to Previous Analysis: Data Segmentation

In the case of a critical phase transition, an increase in temporal autocorrelations and variance is expected near tipping points (Weinans et al., 2021). Some of these phenomena are known to be quantified by RQA measures, a windowed RQA analysis is for example able to detect control parameter changes of the logistic map from periodic-chaos, but also chaos-chaos transitions (cf. Marwan et al., 2009). Especially the expected changes in the geometric structure of the phase space trajectory is expected to be quantified by the RN measures, this occurs when previously inaccessible degrees of freedom become available to the system, while availability of others may be constrained. Note that these assumptions only hold if it is indeed the case that the phase transition is of the critical type and not e.g. a noise induced transition.

**FIGURE 6:**
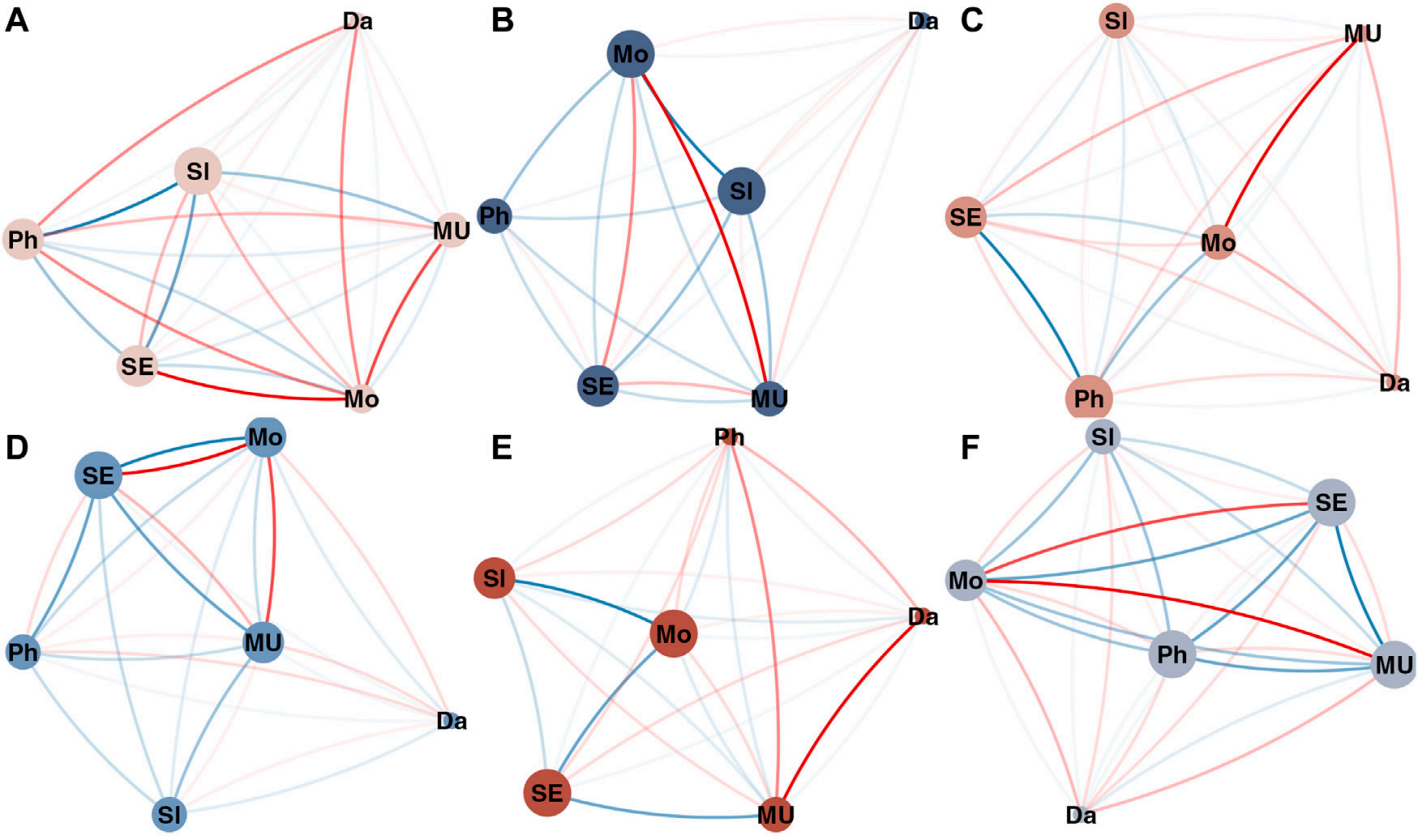
Multiplex cumulative Recurrence Networks for different phases of the experiment: **(A)** Baseline; **(B)** Start double blind; **(C)** Start reduction; **(D)** Post reduction; **(E)** Critical transition; **(F)** Post reduction (additional). Each vertex represents a cumulative Recurrence Network (see Figure 5). The edges represent Inter-layer Mutual information (blue) and Edge Overlap (red). The second part of the post reduction phase after the critical transition is not shown here.

**FIGURE 7:**
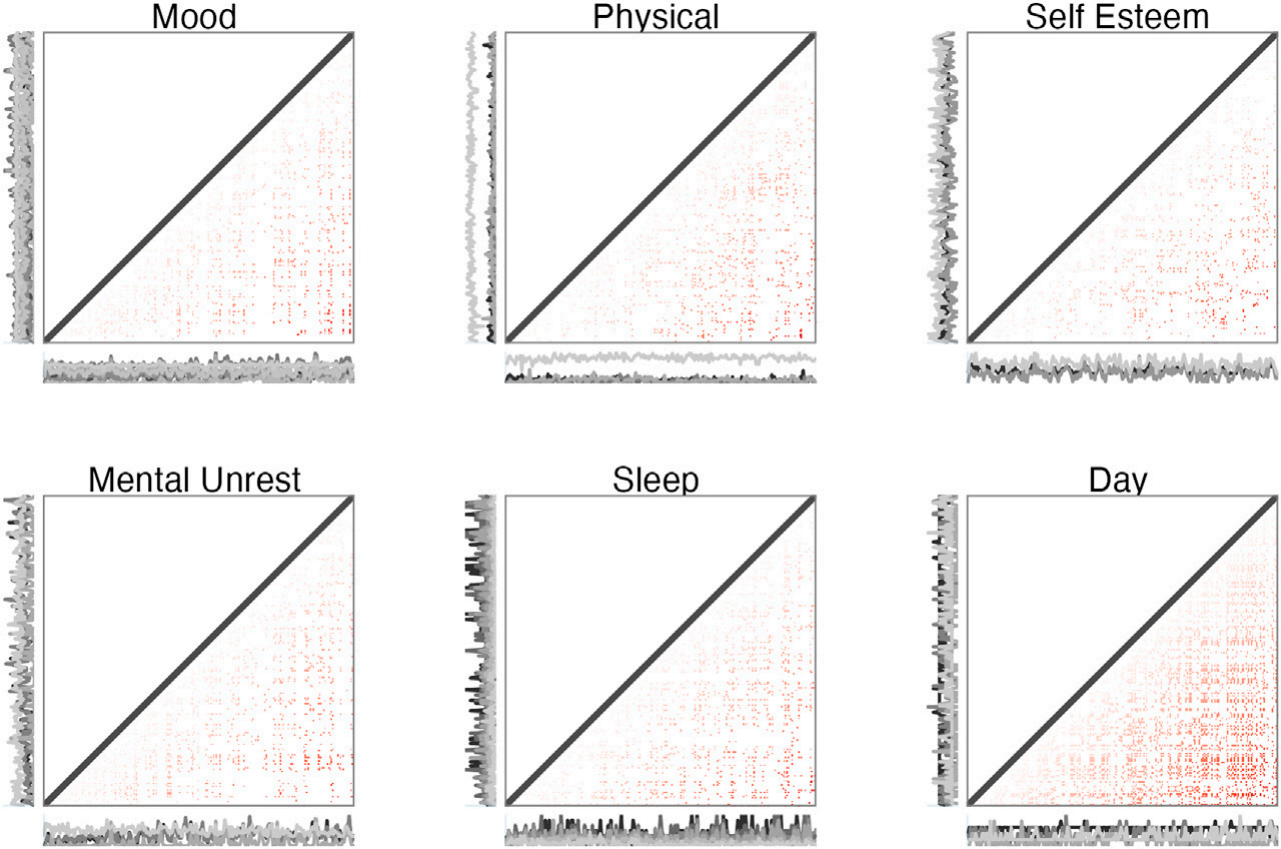
Weighted, directed matrices for constructing cumulative Recurrence Networks of selected variables of the multivariate dataset. The weights represent recurrence times. The direction is implemented by only considering the recurrent points in the lower triangle, and calculating measures based on the out-degree.

Figure 6 is conceptually similar to Figure 1B in the paper by Cramer et al. (2016), which shows the 5-node GGM symptom network fitted to data from different phases of the experiment (data-segmentation). The purpose of constructing a GGM for the different phases of the experiment prior to the major shift in depressive symptoms was to evaluate whether the graph structure would represent the hypothesized increase in correlation between different symptoms. Cramer et al. (2016) indeed found this at the level of the aggregated dimensions *mental unrest*, *positive affect*, *negative affect*, and vertices based on single time series *worry* and *suspicious*. The graphs displayed in Figure 6 are Multiplex cumulative Recurrence Networks based on six *ɛ*-cRN, constructed from distance matrices that represent a phase space composed of subsets of the multivariate data (see the matrices in Figure 7). The Inter-layer MI (blue) and Edge Overlap (red) are shown as edge weights (the edges with lower weights are more transparent to make structural differences more visually apparent). Table 3 displays the average Inter-layer MI (which is the same as the average Edge Strength Density), the average Inter-layer Correlation, the average Edge Overlap and the Global Efficiency of the McRN for the different phases of the experiment. For each measure, it is the case that the McRN of the epoch marked as *critical transition* has the highest value relative to other phases of the experiment.

**TABLE 3:** The Average Edge Strength Density, Global Efficiency, Average Inter-layer MI and Average Edge Overlap of the Multiplex Recurrence Network based on Different Phases in the Experiment.

	Edge Strength	Efficiency	MI	R	Edge Overlap
	$\left\langle \rho_{s}\left(\varepsilon \right)\right\rangle$	$\hat{E}\left(\varepsilon \right)$	$\left\langle I_{\alpha ,\beta }\right\rangle$	$\left\langle R_{\alpha ,\beta }\right\rangle$	$\left\langle \omega \right\rangle$
baseline [N = 28]	0.24	0.18	0.24	0.06	0.06
start double blind [N = 14]	0.19	0.11	0.19	0.02	0.06
start reduction [N = 56]	0.03	0.02	0.03	0.30	0.07
post reduction 1 [N = 29]	0.09	0.07	0.09	0.06	0.05
critical transition [N = 10]	0.31	0.28	0.31	0.15	0.10
post reduction 2 [N = 15]	0.24	0.22	0.24	0.18	0.04
post reduction (additional) [N = 91]	0.01	0.01	0.01	0.25	0.06

Note: Values were calculated on different number of data points [N].

**FIGURE 8:**
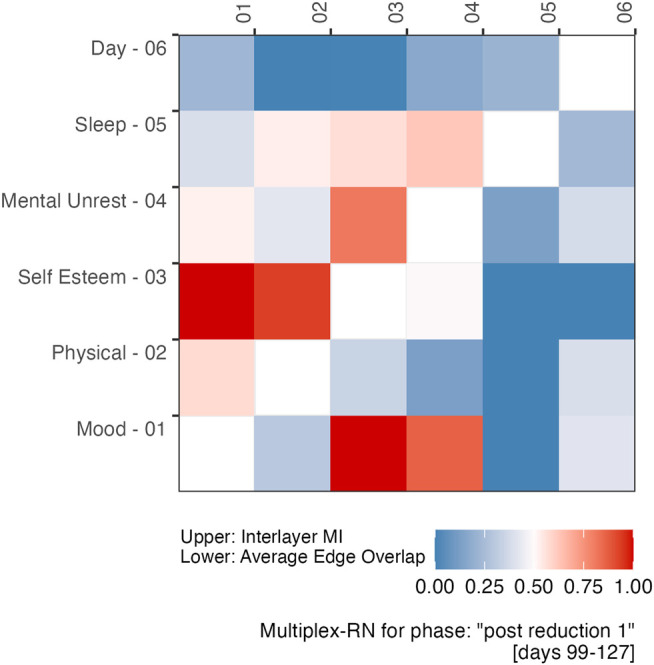
An example of a distance matrix from which a Multiplex Recurrence Network can be constructed. The upper triangle is based on the Inter-layer Mutual Information, the lower triangle represents the Average Edge Overlap between network layers. The values were transformed to the unit scale, using the maxima and minima observed during the post reduction period.

In addition to the fact that the analysis strategy by Cramer et al. (2016) can only be conducted post data collection, another problem is that it is not possible to “de-aggregate” their mental-unrest and affect dimensions to study which variables might be driving the evolution towards the regime shift. The same holds for the windowed analysis of the autocorrelation and variance, based on the summed, detrended score of the five mental states and a window of 30 days. At the level of the multiplex network, one can examine the vertex properties of each individual layer, for example centrality. An example is displayed in Figure 8: The upper triangle of the matrix is a representation of the weighted matrix of the McRN based on Inter-layer MI shown in Figure 6 for the period “post reduction (planned),” the lower triangle displays an weighted matrix based on the Edge Overlap for the same period.

### Geometric Resilience Loss Indicators

Cramer et al. (2016) evidenced critical slowing down by means of a windowed analysis of the variance and the autocorrelation function in a window of 30 days. An increase in the lag-1 autocorrelation and variance was observed with the autocorrelation reaching a peak just before the critical transition. The variance steadily increased during the observation period. A window of 30 days would not be a practical time period for detecting EWS in a clinical setting. Moreover, a closer inspection of the state variable suggests that there are more sudden changes in symptom severity than the one which caused a temporary halt on the measurement of some of the variables starting at day 127.

**FIGURE 9:**
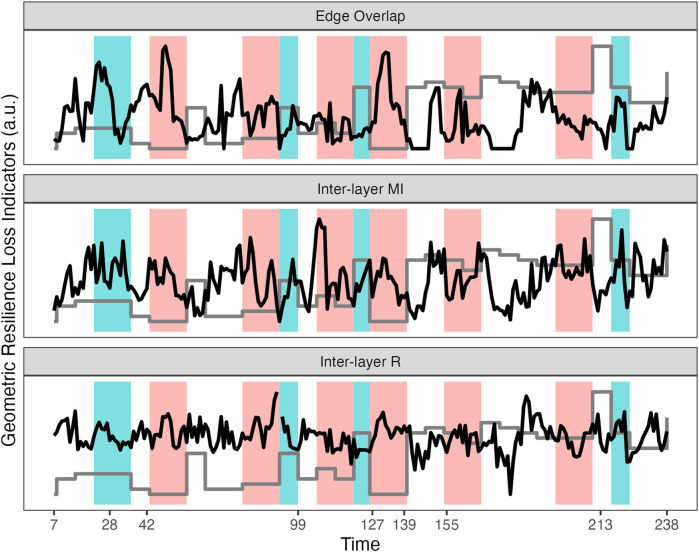
The time course of the Multiplex Recurrence Network measures calculated in a 7 days sliding window: average Edge Overlap, strength-based Inter-layer Mutual Information, strength based Inter-layer degree correlation R. All values are transformed to the unit scale. The grey line represents the major shifts in the global state variable, the depression symptom scale SCL-R-90 which was measured weekly. The colored regions represent a seven or 14 day period before an major change in symptom severity.

Figure 9 displays sliding window analyses (7 days window) of three layer-similarity measures: Edge Overlap, Strength-based Inter-layer Mutual Information, strength based Inter-layer Correlation. The red and green areas represent a period of 14 days before a major change in the state variable (an increase or decrease in symptom severity respectively) occurred. Visual inspection shows that major shifts in symptom severity are often preceded by clear peaks in the Edge Overlap. The Inter-layer MI is similar to the Edge Overlap, but less clear, for the correlation between the strength distributions of the layers it is difficult to assess clear peaks. There are also shifts in symptom severity without any, or, less pronounced peaks preceding it.

**FIGURE 10:**
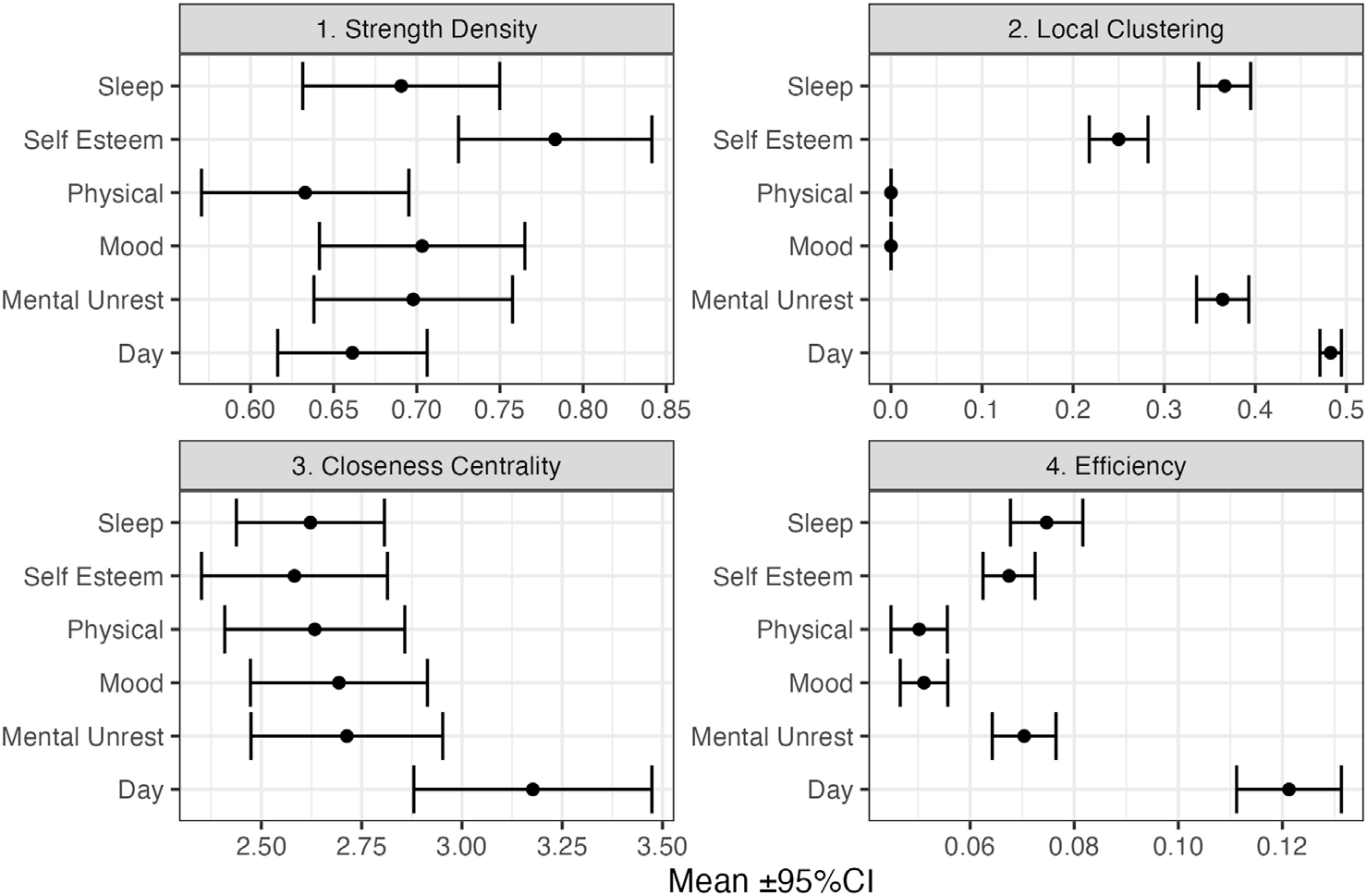
Mean Local vertex characteristics over all 7-days windows (*N* = 232) for each layer in the Multiplex cRN. The figure displays the results based on the MRN with inter-layer MI as edge weights.

The average Edge Overlap (as well as Inter-layer MI and R) can be considered a global network measure of coherence between the layers of the multiplex. In the present context of EWS, the fact a peak is observed before transitions is similar to the hypothesized increase in autocorrelation associated with critical slowing down, which can also be evidenced by performing a PCA on multivariate time series data and observing that most variables load on the principle component with the highest eigenvalue (cf. Weinans et al., 2021). Figure 10 show the average of local network measures across all 7 days windows, for each subsystem separately. Several observations can be made, it appears that the layers “Day” and “Self Esteem” often play a central role in the multiplex network, as indicated by their relatively high mean values for Clustering, Closeness and Efficiency. In the context of recurrence networks, Clustering (and Strength Density) are indicative of the geometric alignment of the state vectors which can occur close to dynamic invariant structures in phase space (Zou et al., 2019). Closeness Centrality and Efficiency represent measures of the shortest path lengths between vertices where the Efficiency measure is less affected by disconnected networks.

## Discussion

The goal of the present paper was to examine the potential of recurrence based analyses as a method for use in a clinical context in which process monitoring of physical and mental states is used to detect early warning signals of imminent shifts in a state variable, for example, the severity of depressive symptoms. The analysis strategy focused on analyzing only those data points that would be available to a clinician in real time, using sliding window analyses with a window size that would be acceptable in setting in which a patient receives an intervention (7 days).

**FIGURE 11:**
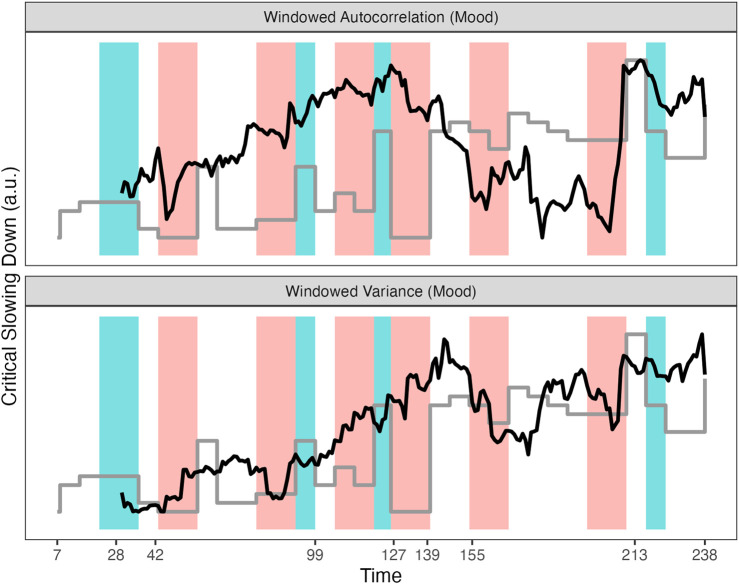
Variance and Autocorrelation of the variables in the Mood subsystem calculated in a sliding window of 30 days. The grey line represents the depression symptom scale SCL-R-90. See text for details.

The analyses reveal that layer similarity measures of a Multiplex network of which the layers consist of cumulative (directed and weighted) recurrence networks display peaks within a 7–14 days window before a major shift occurred in the state variable. Local network measures were examined and revealed two subsystems, “Day” and “Self Esteem,” which played a central role in the Multiplex network. The peaks in the layer similarity measures are most clearly visible in the Edge Overlap and Inter-layer Mutual Information. However, not every transition is preceded by a peak, and not every peak indicates a transition is imminent within 7–14 days. For comparison, see Figure 11 which approximately mimics the original analysis by (Wichers et al., 2016) evidencing Critical Slowing Down in the windowed autocorrelation and variance of the Mood subset of variables. There are several reasons why the measures in Figure 11 are not suitable for the goals set for the present study. First, a 30 days window was used which is not practical for most clinical settings. Second, the autocorrelation has its highest peak at exactly the moment of the transition (day 127). Third, the windowed variance actually increases after the transition, and reaches its highest peak after the experiment was re-started. Fourth, there are no apparent associations between peaks in these measures and other major transitions in the state variable.

**FIGURE 12:**
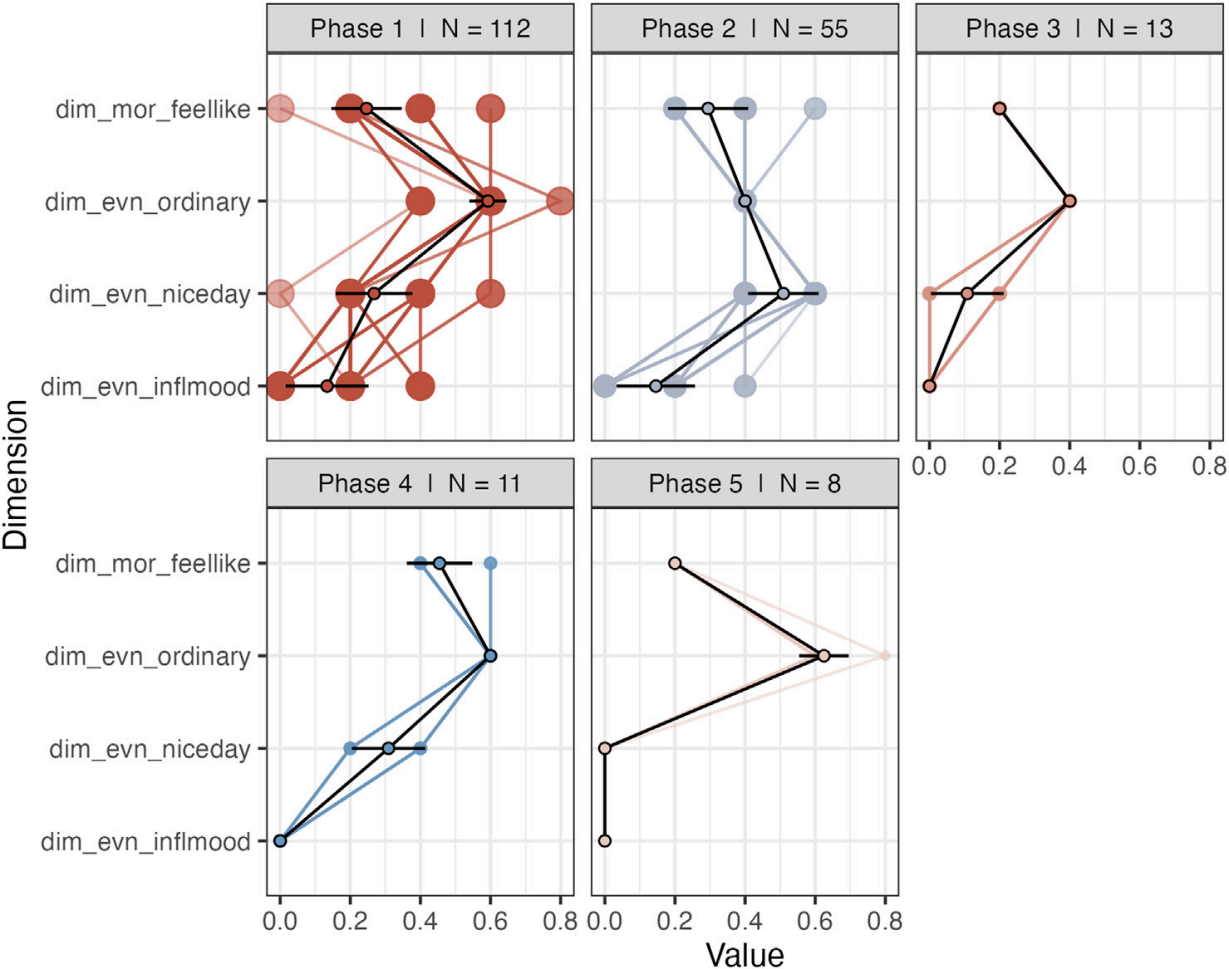
Profiles of recurring phases of the Day subsystem.

**FIGURE 13:**
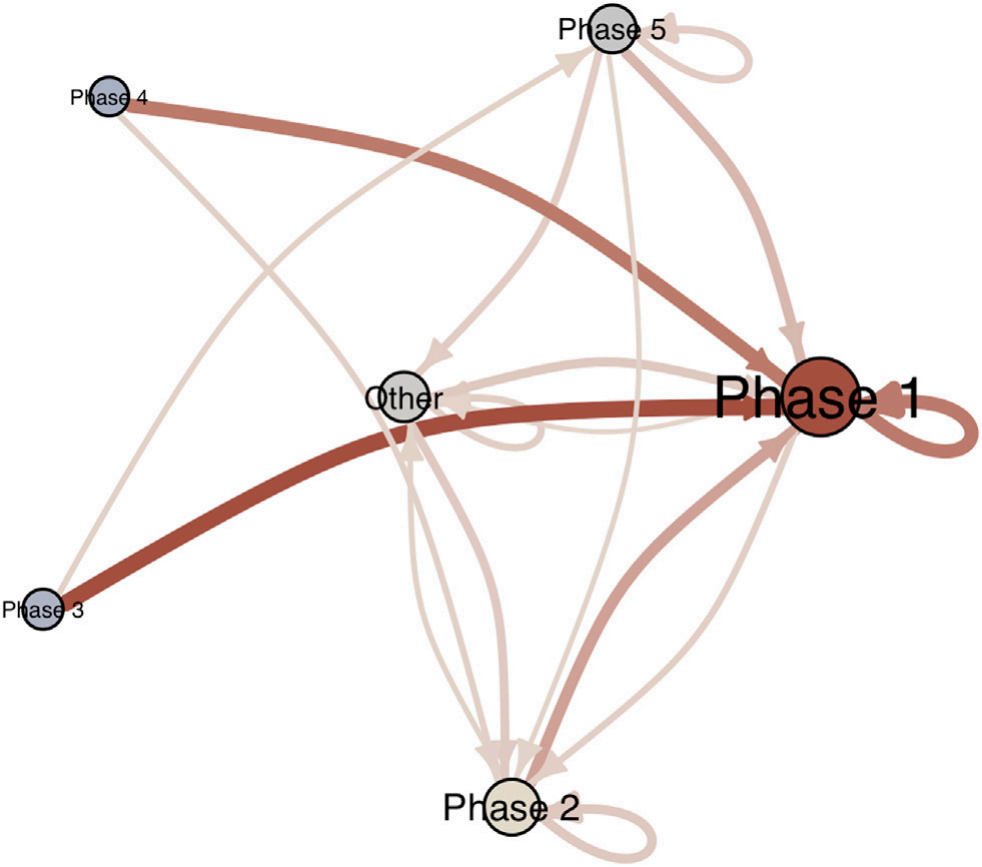
Transition Network of the phases of the subsystem Day. Edges with a transition probability below 0.10 are not displayed.

An advantage of the current analysis strategy over the original study is that there is no aggregation of the multivariate time series for the purpose of dimension reduction. That is, it is possible to go back to the individual dimensions to further investigate the profiles of recurring phases (coordinates in phase space) and construct a transition matrix (see Heino et al., 2021). Figure 12 displays the profiles of states in the “Day” subsystem, sorted from most-to-least frequently recurring. The most frequently recurring phase can be characterized as a mundane experience of the day (high on ordinary day, neutral to positive on prospective and reflective evaluation of the day)^1^. The second phase is different due to the response to “I found this a nice day,” which is now in the direction of strong disagreement. The other phases are characterized by higher disagreement with “looking forward” to the day. This provides a detailed window into phase space dynamics that would have obviously been lost if the six series of the Day subsystem were aggregated into 1 time series. Based on the sequences of phases it is possible to construct a transition matrix and the corresponding directed graph is displayed in Figure 13. The transition network can reveal insights into the existence of certain paths that frequently lead to certain phases. In Figure 13 it is clear that Phase 1 and Phase 2 are highly connected, but Phase 1 is the default state, to which the system most frequently returns.

### Limitations and Future Directions

As can be seen in Figure 9 it is often the case a peak in the daily measurements precedes a shift in the weekly observed symptom severity, but not in every case and not for every measure examined. There are several explanations, for example, the symptom severity scale was observed less frequently and fluctuations occurring within a week were not observed by design. A solution could be to consider a (set of) daily measured variables to represent the state variable, but there are also benefits to using a diagnostic scale (e.g., norms exist). Perhaps a more dynamic test could be developed and normed, one which allows for daily assessment of symptoms (e.g., by creating several parallel tests).

Another limitation of the method as presented in this paper is the lack of a reference for determining whether a peak is a potential EWS or merely a random fluctuation. There are several ways to at least check whether the observed peaks are “real”, for example, by repeating the analysis several times on surrogate data (e.g., Jacob et al., 2018) or perform a block-randomization on the windowed time course (e.g., Vink et al., 2018). Another way to construct a randomization test could be by randomly re-wiring the network layers and re-construct the Multiplex network and evaluate and compare the layer similarities to the observed measures.

A related issue concerns the fact that EWS are naive to the direction of change, that is, to be of clinical use it would be helpful to know whether an EWS implies the current state is deteriorating (higher symptom severity) or not. There have been theoretical advances to finding the direction of least resilience in multivariate time series data (Weinans et al., 2019) or based on formal models (Cui et al., 2021). Recently Schreuder et al. (2022) used a technique based on two variables obtained from windowed principal component analysis (the variance explained by the first component and the skewness of the scores projected onto this component) to identify the direction of the critical transition in the same data set used in the present paper. In the same study, the method was confirmed to predict the direction of transition in a larger sample of 34 individuals. Further study is needed to evaluate whether the technique can be used in a clinical setting according to the goals set for the purpose of the current paper. The EWS phenomenon evidenced by Schreuder et al. (2022) (uni-dimensional behavior of system dimensions before a critical transition), is very similar to the phenomena indicated by the geometric recurrence network measures used in the present study: Layers in the McRN become structurally more similar (MI or correlation between degree distributions, edges connecting to the same vertices) and the local centrality measures indicate geometric alignment of the phase space vectors.

To summarize, recurrence network analysis of multivariate time series data has been shown to have great potential for use in clinical practice, specifically in the context of real-time process monitoring of individual patients. The current example has been a proof of principle, more thorough study and development is required into the relationship between transitions and the behavior of the (multiplex) recurrence measures.

## Data Availability

The original contributions presented in the study are included in the article/Supplementary Material, further inquiries can be directed to the corresponding author. The data from Wichers et al. (2016) were published in the Journal of Open Psychology Data (https://openpsychologydata.metajnl.com/articles/10.5334/jopd.29/) and are publicly available at: https://osf.io/j4fg8 unde a CC-By Attribution 4.0 International (CC-By) License. The analysis scripts used in this paper are available at: https://osf.io/5947r.

## Footnotes

1 The questions were: “I found this an ordinary day,” “I found this a nice day” (recoded), “Filling in this questionnaire influenced my mood,” “I am looking forward to this day” (recoded).
